# Early loading of plalatal implants (ortho-type II) a prospective multicenter randomized controlled clinical trial

**DOI:** 10.1186/1745-6215-8-24

**Published:** 2007-09-20

**Authors:** Britta A Jung, Heinrich Wehrbein, Werner Hopfenmüller, Winfried Harzer, Tomasz Gedrange, Peter Diedrich, Martin Kunkel

**Affiliations:** 1 Department of Orthodontics, University Hospital Mainz, Augustusplatz 2, 55131 Mainz, Germany; 2 Institute for Medical Statistics, Charité-Campus Benjamin Franklin, University of Berlin, Hindenburgdamm 30, 12203 Berlin, Germany; 3 Department of Orthodontics, Technical University of Dresden, Fetscherstr. 74, 01307 Dresden, Germany; 4 Department of Orthodontics, University of Greifswald, Rotgerberstr. 8, 17487 Greifswald, Germany; 5 Department of Orthodontics, University of Aachen, Pauwelsstr. 30, 52074 Aachen, Germany; 6 Department of Oral and Maxillofacial Surgery, University Hospital Mainz, Augustusplatz 2, 55131 Mainz, Germany

## Abstract

**Background:**

In orthodontic treatment, anchorage control is a fundamental aspect. Usually conventional mechanism for orthodontic anchorage control can be either extraoral or intraoral that is headgear or intermaxillary elastics. Their use are combined with various side effects such as tipping of occlusal plane or undesirable movements of teeth. Especially in cases, where key-teeth are missing, conventional anchorage defined as tooth-borne anchorage will meet limitations. Therefore, the use of endosseous implants for anchorage purposes are increasingly used to achieve positional stability and maximum anchorage.

**Methods/Design:**

The intended study is designed as a prospective, multicenter randomized controlled trial (RCT), comparing and contrasting the effect of early loading of palatal implant therapy versus implant loading after 12 weeks post implantation using the new ortho-implant type II anchor system device (Orthosystem Straumann, Basel, Switzerland).

124 participants, mainly adult males or females, whose diagnoses require temporary stationary implant-based anchorage treatment will be randomized 1:1 to one of two treatment groups: group 1 will receive a loading of implant standard therapy after a healing period of 12 week (gold standard), whereas group 2 will receive an early loading of orthodontic implants within 1 week after implant insertion. Participants will be at least followed for 12 months after implant placement.

The primary endpoint is to investigate the behavior of early loaded palatal implants in order to find out if shorter healing periods might be justified to accelerate active orthodontic treatment. Secondary outcomes will focus e.g. on achievement of orthodontic treatment goals and quantity of direct implant-bone interface of removed bone specimens. As tertiary objective, a histologic and microtomography evaluation of all retrieved implants will be performed to obtain data on the performance of the SLA surface in human bone evaluation of all retrieved implants. Additionally, resonance frequency analysis (RFA, Osstell™ mentor) will be used at different times for clinically monitoring the implant stability and for histological comparison in order to measure the reliability of the resonance frequency measuring device.

**Trial registration:**

Current Controlled Trials ISRCTN97142521.

## Background

Control of anchorage for orthodontic tooth movements represents a fundamental problem in the treatment of dental and skeletal dysgnathia. Available anchorage potential [1,2], and that required for each anchoring task, must be taken into consideration if undesired tooth movements and anchorage loss are to be avoided. To avoid uncontrolled tooth movements, the existing anchorage potential of the natural teeth has to be balanced to the required anchorage task. This may pose a clinical challenge when strict positional stability and maximal anchorage is intended.

According to these general rules, a reduced number of anchor teeth, advanced loss of periodontal attachment or an unfavourable distribution of teeth represent the typical clinical findings to consider skeletal anchorage [3]. When traditional anchorage concepts like the Nance button appliance or a lingual arch or even extraoral (headgear or Delaire face mask) devices are applied in these demanding cases, they often produce unpredictable reactive forces and moments [4,5]. Specifically, they often lead to protrusion of the incisors, extrusion and tipping of the anchoring teeth, and interfere with the alignment of the occlusal plane.

For these reasons, temporary orthodontic anchorage implants were developed for the maxilla in the early 1990s. Usually, according to the present manufacturers' instructions and to conventional loading concepts in general dental implantology, palatal implants are loaded after a healing period of 3 (-4) months. Recently, improvements in general implant design and surface, which have increased bone-to-implant contact rates, have encouraged changes in conventional loading protocols in favour of early and immediate loading concepts [6-8].

According to experimental and clinical literature and to previous consensus statements [9,10], reduced implant healing times can be recommended in many implant indications. However, comparative clinical trials concerning immediate and early loading of palatal implants used for maximum anchorage are rare at present [11].

Therefore, this study will focus on early implant loading using the new ortho-implant type II anchoring device (Orthosystem^®^, Straumann, Basel, Switzerland) to investigate if shorter healing periods might be justified in order to accelerate orthodontic treatment.

## Methods/Design

### Study design

The study is designed as a prospective, multicenter randomized controlled clinical trial. The study was approved by the Ethics Committee of the State Medical Council of Rhineland-Palatinate, Germany (Ref. No: 837.210.06 (5308)).

A formal coordination center for clinical trials (KKS Mainz, Germany) will monitor study progress and assure data accuracy.

### Study objectives

The objective of the clinical study proposal will be to investigate the performance of early functional palatal implant loading in order to find out if:

- early orthodontic loading without the typical healing period is a clinically safe procedure

- and might, thus, be justified to accelerate active orthodontic treatment.

Therefore, the study will compare and contrast the efficacy and results of early functional implant loading within 1 week versus conventional implant loading after 12 weeks post implantation.

Concerning this matter the following null hypothesis will be addressed: There will be no difference between standard therapy and early loading group concerning implant failure rate.

### Patients

Males or females whose diagnoses require temporary stationary implant-based orthodontic anchorage treatment will be included in the study. Inclusion criteria for the study are as follows:

• Orthodontic indication for skeletal anchorage

• Adequate bone quantity for a palatal implant in the lateral cephalogram

• Good oral hygiene and normal wound healing capacity

• Written informed consent

Patients with cheilognathopalatoschisis and other syndromes associated with craniofacial anomalies are to be excluded. Other exclusion criteria are patients with immunodeficiency, diseases requiring a prolonged steroid usage, previous radiation therapy or chemotherapy, patients with metabolic bone diseases or uncontrolled endocrine disorders, alcohol or drug abuse as well as pregnancy.

### Study interventions

Implant loading after a post-surgical healing period of 12 weeks (standard therapy, group 1) versus early implant loading within 1 week post implantation (group 2).

### Study device

**Figure 1:**
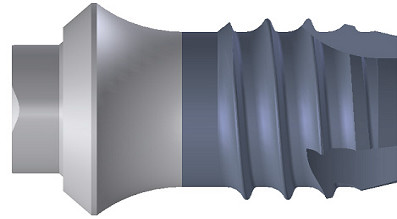
Schematic representation of an orthodontic anchorage implant (ortho-type II, Straumann, Basel, Switzerland). The Ortho-type II implant is available in the dimensions 4.1 mm (4.8 mm) × 4.2 mm.

The new ortho-implant type II anchor system (Orthosystem^®^, Straumann, Basel, Switzerland; Fig.1) available with diameters of 4.1 mm and 4.8 mm, is a pure titanium 1-piece device. It consists of a endosseous implant body, a transmucosal implant neck, abutment, fixation cap and occlusal screw, all parts being made of pure titanium except the occlusal screw (stainless steal). The endosseous implant body has a self-tapping thread with a sandblasted, large grit, acid-etched (SLA^®^) surface. A set of burs and instruments is available for implant insertion and removal. For orthodontic treatment the implant will be connected to a rotationally secure steel abutment coping (length: 3.6 mm; diameter: 5.0 mm), onto which orthodontic arches are fixed in position by laser welding.

### Implant Insertion

Cephalometric analysis of lateral radiographs must be available to determine the optimal implant insertion site. Implants will be inserted in the paramedian or median palatal area to obtain good primary stability and maximum bone integration of the implant surface.

Preoperative antibiotic prophylaxis will be given before implant's insertion. Surgical placement of the implant in the maxillary anterior area will be done under local anesthesia (palatine and incisal nerves) according to the manufacturers' instructions [12]. Oral hygienic instruction must be given by the surgeon comprising the use of chlorhexidine digluconate solution.

Lateral radiograph must be performed post surgery in order to verify the correct position and angulation of the implant.

### Sample Size

For sample size calculation an implant failure rate of 5 % for group 1 (implant loading after a post-surgical healing period of 12 weeks) an implant failure rate of 25 % for group 2 (early implant loading within 1 week post implantation) was assumed. Based on 0.8 power to detect a significant difference at the two sided 5% level with an assumed loss to follow-up of 5 %, 62 patients in each group and 124 in total will be required. Sample size calculation, which based on clinical experiences with the new Orthosystem type II and early functional loading of conventional dental implants, was estimated with nQuery Advisor^® ^(Version 3.0) by the Institute of Medical Biostatistics, Epidemiology and Informatics (WH), University of Berlin, Germany.

### Randomization

At each center, investigators and participants will be kept from knowing which patient will be assigned to which treatment group until post implant insertion. When according to the surgeon intraoperatively implant primary stability is given, participants will be randomly assigned either to the control or experimental group. Randomization will be performed in a 1:1 ratio using a balanced design with a block of 4 to treatment based on a computer-generated randomization code by an Institute of Medical Biostatistics (KKS, Mainz, Germany).

### Orthodontic treatment and superstructure

Postoperative checkups will be completed after one, two, six and twelve weeks respectively. The patients will be instructed to rinse with a chlorhexidine digluconate solution in the first ten days postoperatively.

**Figure 2:**
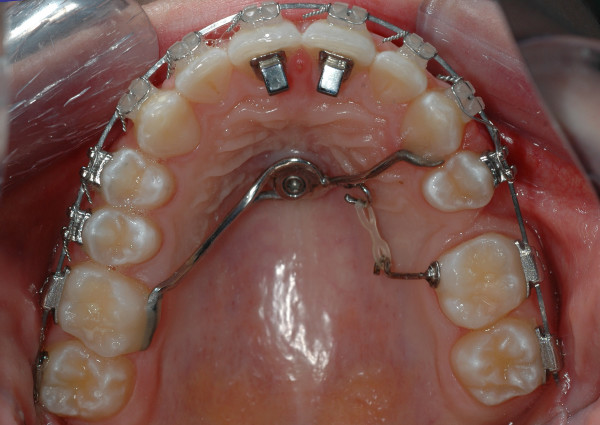
Intraoral photograph of the upper jaw showing indirect implant anchorage (rigid connection between the anchorage teeth and implant) using a modified transpalatal arch.

An impression will be taken of the implant with alginate and preformed caps and a model fabricated. A custom-designed palatal superstructure will be fabricated on the working model depending on malocclusion type and biomechanics requirements (Fig. 2).

Active orthodontic treatment starts for group 1 (implant loading after 12 post-surgical weeks) when the osseointegration period is completed and the implant shows good secondary stability. Active orthodontic treatment for group 2 (early implant loading) starts within 1 week after implant insertion, when the implant shows a good primary stability and ends after completion of active treatment.

### Outcome measurements

#### Primary Clinical Endpoints

Implants' stability after a loading time of 6 and 12 months of function after implant placement. The criteria of implant success are:

- implant survival

- no abnormal mobility

Implant stability will be tested post implant placement and throughout the orthodontic controls using the percussion test. Therefore the palatal implant in each case will be tapped then in vertical and horizontal directions using an instrument grip to test the subsequent resonance on percussion. High acustic sound response will indicate good primary and secondary implants' stability respectively, whereas dull acustic sounds will indicate loss of implants' stability.

In addition, resonance frequency analysis (RFA, Osstell™ mentor) will be used at time of surgery and at different times during postoperative checkups and finally after completion of active treatment. Resonance frequency analysis, firstly introduced by Meredith [13], is a method for clinically monitoring the implant stability in implant stability quotient (ISQ) units, which is scaled from 1 to 100. For measuring implant stability the orthodontic suprastructures will be removed.

#### Secondary Clinical Endpoints

Secondary clinical endpoints will include achievement of partial orthodontic treatment success 12 months after implant insertion, quantity of direct implant-bone interface of the removed bone specimens, patient's acceptance rate of palatal implants, anchorage loss of the anchor tooth unit and overall success after completion of active treatment.

Therefore, the set of model casts before and after 12 months' treatment will be compared in order to evaluate the partial orthodontic treatment success based on the peer assessment rating (PAR) index. The PAR index is an occlusal index, especially designed and validated as an objective instrument to measure how much the dentition of a patient deviates from normal occlusion. The index scores maxillary and mandibular alignment (crowding and spacing), buccal segment occlusion (anterioposterior, transverse and vertical), overjet (including anterior crossbite), overbite, and midline discrepancies. The more points the more severe is the malocclusion. Additional measurements such as the percentage of space closure in extraction cases, or the percentage of molar distalization will be taken.

The entire set of model casts before and after treatment will also be compared in order to evaluate the overall anchorage loss of the anchor unit, linear changes in the transverse dimension and finally to evaluate the overall treatment success, using the peer assessment rating (PAR) index. Analysis and superimposition of pretreatment and postreatment lateral cephalograms using Pancherz's analysis [14] will be performed to evaluate dental and skeletal changes by linear and angular measurements.

The objectives of the histomorphometric investigation will be to analyse quantitatively the percentage of direct bone-to-implant contact of the explanted orthodontic anchorage implants as well as to measure the distance between marginal bone surface and transmucosal implant neck of the explanted palatal implants in group 1 (implant loading after 12 post-surgical weeks) and group 2 (early implant loading within 1 week post surgery).

To investigate the removed bone specimens histomorphometrically, they will be immersed by ascending concentrations up to absolute ethanole for 2 weeks and will then be embedded in a mixture of glycol methacrylate (Technovit 7200 VLC^®^, Kulzer& Co Ltd., Wehrheim, Germany). Polymerisation will be obtained by light curing with a 450 nm wave length light source. From each specimen, sections parallel to the implant's longitudinal axis will be cut using a diamond band saw followed by grinding and polishing on an EXACT Microgrinding System (EXACT, Norderstedt, Germany) to a final thickness of 20 μm. The sections will be stained by toluidine-blue for transmission light microscopy.

In order to evaluate the patient's satisfaction and tolerance towards an implant-based orthodontic treatment, a questionnaire will be given to all patients at the end of active treatment, irrespective of malocclusion type and biomechanics.

The questionnaire will address the physiological and psychological responses of patients with special regard patient's comfort during an implant-based orthodontic treatment.

#### Tertiary Clinical Endpoints

**Figure 3:**
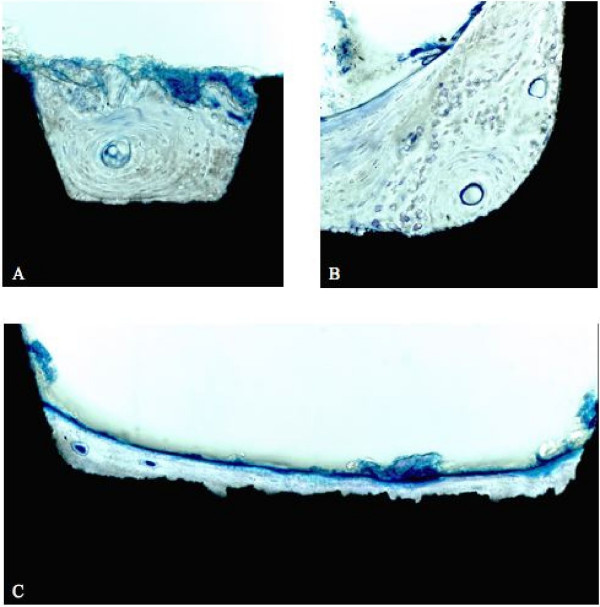
Histological evaluation of SLA^®^-surface performance in human bone. Specimens obtained due to the planned retrieval of Ortho^®^-implants at the end of orthodontic treatment. Bone-to-implant contact in the threaded body (a) and the neck region (b) of explanted human Ortho^®^-implants (Toluidine, original magnification 200 ×). (c) Immediate bone-to-implant contact along the SLA^®^-surface (Toluidine, original magnification 400 ×).

**Figure 4:**
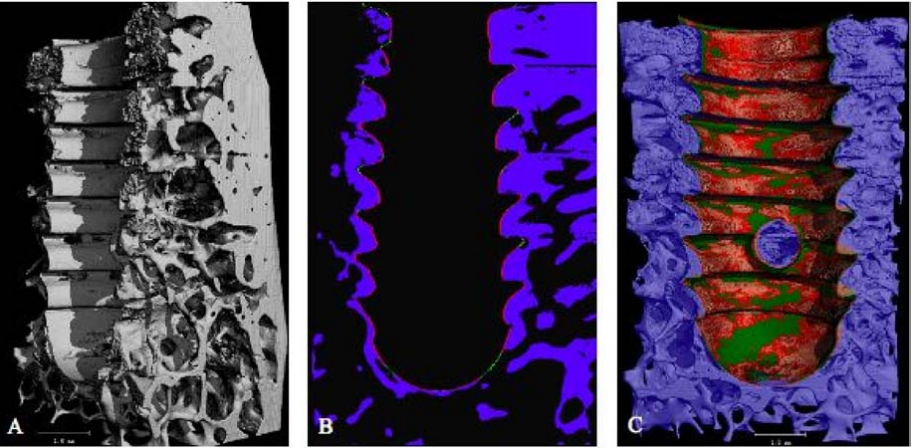
3 D Visualization with Micro-Computed Tomography (Institute of Applied Structure- and Microanalysis (ASMA), University of Mainz). Section of high-resolution micro-computed tomography scan for a retrieved conventional dental titanium implant (a-c). Internal structures are reconstructed as a set of flat cross sections, which were used to analyse the three dimensional morphological parameters of the implant with bone contact zones (a). Blue indicates bone architecture, red shows areas of direct bone-to-implant contact, while green indicates areas of no implant-bone interface (b-c).

As a tertiary endpoint, a histological and microtomography evaluation of all retrieved implants (about 2–3 years after placement) will be performed to obtain data on the performance of the SLA surface in human bone (Fig. 3a–c and 4a–c).

In addition, a comparison between histomorphometric and microtomographic analyses will be performed on each specimen to evaluate the possibility of microtomography and to calibrate the technology for implant osseointegration assessment.

Moreover, the measurement reliability of the resonance frequency measuring device (Osstell™ mentor) in the assessment of implant stability will be evaluated by comparison between ISQ (implant stability quotient) values and the quantity of direct implant-bone interface of all retrieved implants.

Finally, the comparison between histomorphometric and resonance frequency analyses will also be used to define a standardized range of ISQ (implant stability quotient) readings for successful implant osseointegration for palatal implants.

### Statistical analysis

The entry, evaluation, and statistical analysis of all data and measurements are calculated using SPSS^® ^(Statistical Package for Social Science) software for Windows (Chicago, II, USA). Inter alia, the set of data will be analyzed based on implant survival rates.

An interim analysis will be performed for 62 palatal implants 6 months post-surgery. If the results of the interim analysis are an implant failure rate of more than 50 % in any group, the study will be terminated before the planned completion date.

### Timeframe of the study

The overall timeframe for the study is estimated to 5 years (12/2006-12/2011). Patient recruitment period will last 3 years.

## Discussion

Clinical investigations and experimental studies indicate that endosseous implants e.g. palatal implants are resistant to orthodontic force application and can be used to achieve positional stability and therefore to enhance orthodontic anchorage [1,2,4,5,8,11]. Usually, according to the present manufacturers' instructions and to conventional loading concepts in general dental implantology, the healing period for palatal implants must not fall short of 3 to 4 months.

Recent findings from clinical and experimental studies suggested the possibility of loading palatal implants earlier than 12 weeks. Borbély and coworkers [8] showed, that an sufficient amount of osseointegration could be achieved at about 4 weeks after surgery or even at an earlier time. This result was based on clinical and histological evaluation in an experimental animal study (foxhounds). Study endpoints were defined as implant loss and implant stability after 1 and 6 months of loading.

Crismani and coworkers [11] initiated a clinical pilot study to evaluate the behaviour of 20 early loaded palatal implants over an observation time of 12 weeks. They reported a success rate of 90% for implants functionally loaded after a healing period of 1 week. The implants, however, were observed no longer than for the 12 week period.

Therefore, we initiated an RCT study comparing and contrasting the effect of early loading of palatal implant therapy versus implant loading after 12 weeks post implantation using the new ortho-implant type II anchor system device (Orthosystem Straumann, Basel, Switzerland). The results of this trial will be presented as soon as they become available.

## Competing interests

The author(s) declare that they have no competing interests.

## Authors' contributions

BJ and MK designed the study and are the mainly contact persons for the sponsor and secondary study centers. BJ is responsible for study organisation, study documentation including data collection, ethics committee, orthodontic treatment (primary study center) and wrote the manuscript. MK is responsible for surgical implant insertions (primary study center) and histomorphometric analyses. HW has a national and international reputation in skeletal anchorage. His entire experiences and consolidated knowledge concerning skeletal anchorage will be provided for the investigators (BJ, MK, HW, WH, PD, TG) in the present research proposal. Werner Hopfenmüller (WH) performed the sample size calculation. All authors have read and approved the final manuscript.
